# Rare Rotavirus Strains in Children with Severe Diarrhea, Malaysia

**DOI:** 10.3201/eid1705.101652

**Published:** 2011-05

**Authors:** Ling-Sing Ch’ng, Way S. Lee, Carl D. Kirkwood

**Affiliations:** Author affiliations: Murdoch Childrens Research Institute, Parkville, Victoria, Australia (L.-S. Ch’ng, C.D. Kirkwood);; University of Melbourne, Melbourne, Victoria, Australia (L.-S. Ch’ng, C.D. Kirkwood);; University of Malaya, Kuala Lumpur, Malaysia (W.S. Lee)

**Keywords:** rotavirus, diarrhea, children, G3[9], viruses, Malaysia, letter

**To the Editor:** We report the identification of G3P[9] rotavirus in children with acute diarrhea in Malaysia. Globally, rotavirus infections are the most common cause of severe diarrhea in infants and young children admitted to hospital. It is estimated that 527,000 children <5 years of age die each year of rotavirus diarrhea (*1*). Strains with a G3P[9] genotype represent a rare group of viruses, initially reported in Japan in 1982. These viruses have been sporadically associated with diarrhea in infants in countries such as Thailand, Italy, United States, Japan, Malaysia, and China (*2**–**7*) and thus represent a rare but widely distributed group of viruses.

Four genotype G3P[9] strains were identified from a total of 134 rotavirus-positive samples analyzed during surveillance studies conducted among children <5 years of age who were admitted to the University of Malaya Medical Centre, Kuala Lumpur, with acute diarrhea during 2008. To understand the possible origin of these G3P[9] viruses, we determined the sequence of the genes encoding the 2 outer capsid proteins, viral protein (VP) 7 and VP4, and analyzed their phylogenetic relationship to other rotaviruses.

Rotavirus double-stranded RNA was extracted by using the QIAamp Viral RNA Mini Kit (QIAGEN, Hilden, Germany), and the genes encoding the VP4 and VP7 proteins were amplified by reverse transcription–PCR (RT-PCR). The VP7 gene segment (nt 51–932) was amplified by using primers VP7-F and VP7-R (*8*), and the VP8 subunit of the VP4 gene (nt 150–795) was amplified by using the primers VP4-F and VP4-R (*9*). The PCR products were purified by using the QIAquick Gel Extraction Kit (QIAGEN) and sequenced by using the ABI Prism BigDye Terminator cycle sequencing kit version 3.1 (Applied Biosystems, Carlsbad, CA, USA) with primers homologous to both ends and internal regions of each gene. Sequencing was performed on an Applied Biosystems 3730*xl* DNA Analyzer at the Australian Genome Research Facility. Sequences were analyzed by using the Sequencher program version 4.1 (Gene Codes Corp., Inc., Ann Arbor, MI, USA), and aligned by using ClustalW (www.ebi.ac.uk/clustalw). Phylogenetic analysis was conducted by using MEGA version 4.1 and neighbor-joining method with 1,000 bootstrap replicates (*10*).The 4 G3P[9] rotavirus strains all exhibited identical nucleotide and amino acid sequences for the regions of VP7 and VP8 subunit of VP4 analyzed.

The VP7 gene from the G3P[9] strains from Malaysia exhibited greatest identity to VP7 genes from animal G3 rotaviruses; identities were 98% and 97% to a raccoon dog rotavirus (RAC-DG5, Japan, 2004) and a feline rotavirus (Australia, 1984), respectively. Comparison with the prototype G3P[9] strain AU-1 exhibited 90% nt homology. Notably, the VP7 gene of the Malaysian G3P[9] strain also shared 90% nt homology with human G3 strains isolated in Malaysia in 2004 and 2007. Phylogenetic analysis of the VP7 nucleotide sequence (nt 93–877) revealed that the Malaysian G3P[9] strain (552157) clustered with animal G3 strains and human G3P[9] strains from various countries but was distinct from G3P[8] strains causing disease in children in Malaysia over the same period (Figure, panel A).

Similar to the VP7 gene, the VP8 subunit of VP4 of the G3P[9] strains from Malaysia exhibited greatest nucleotide homology (98%) with the rotavirus strain isolated from a raccoon dog (RAC-DG5). High nucleotide homology of 96%–98% was also observed when the P[9] strain from Malaysia was compared with other human P[9] rotavirus strains isolated in Japan, Thailand, and China. Phylogenetic analysis revealed 3 distinct clusters among the VP8 sequences obtained from the P[9] strains (Figure, panel B). Human and animal P[9] strains from Asia grouped together within a single cluster. The feline strains from Italy and Australia grouped together, as did the P[9] strains from the United States.

**Figure:**
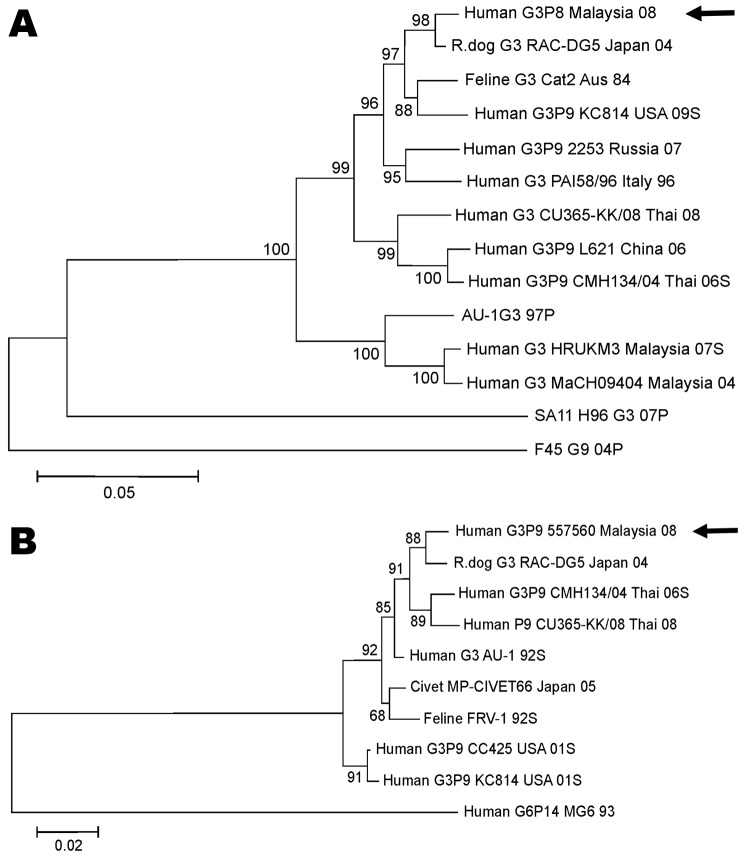
Phylogenetic relationship of nucleotide sequences of genes encoding the outer capsid proteins VP7 and VP4 from G3P[9] rotavirus strains. A) Evolutionary relationship of G3 VP7 nucleotide sequences. B) Evolutionary relationship of P[9] VP4 nucleotide sequences. The evolutionary relationship was inferred by using the neighbor-joining method. The percentages of the bootstrap test (2,000 replicates) are shown next to the branches. The evolutionary distances were computed by using the maximum-composite likelihood method and represent the number of base substitutions per site. Phylogenetic analysis was conducted by using MEGA version 4 (*10*). The labeling of the taxon corresponds to host name, followed by G-type and/or P-type, strain name, place of origin, and year of isolation. The letter S or P after the year of isolation indicates submission or published year of the sequence in the National Center for Biotechnology Information database. Arrows indicate G3P[9] isolate identified in this study. Scale bars indicate nucleotide substitutions per site.

Thus, both the VP7 and VP4 genes of G3P[9] strain identified in this study were most closely related to a racoon dog rotavirus strain (RAC-DG5), suggesting an animal origin of this rotavirus strain. These strains are likely an example of an animal strain causing limited disease in humans, rather than existence of a true strain, which has entered and adapted to the human environment. Recent whole-genome sequencing of 2 G3P[9] strains isolated from children in Italy showed they were composed of genes of human, bovine, and feline origin (*2*); whether the G3P[9] strains from Malaysia identified in this study are also human/animal reassortant strains requires further study.

Identification of G3P[9] strains in Malaysia continues to highlight the presence of these rare strains in Asian communities. The close similarity of the strains to a G3P[9] strain from a raccoon dog further highlights the transmission of rotavirus strains between animal and human sources. Whether this strain can establish itself in humans and cause disease is unknown, but the identification of rare strains illustrates that movement of rotaviruses between various hosts does occur from time to time.
